# *Mycobacterium tuberculosis* hijacks the UBE2O pathway to regulate host iron homeostasis

**DOI:** 10.1172/JCI184095

**Published:** 2025-05-01

**Authors:** Tran Xuan Ngoc Huy, Huynh Tan Hop

**Affiliations:** 1 Institute of Animal Medicine, Gyeongsang National University, Jinju, South Korea,; 2 University Center for Bioscience and Biotechnology, National Cheng Kung University, Tainan, Taiwan.

**Keywords:** Immunology, Infectious disease, Microbiology, Bacterial infections, Macrophages

To the Editor: Mycobacterium tuberculosis is a causative agent of tuberculosis, which causes approximately 1.5 million deaths annually. An essential virulence strategy relies on mycobacterial survival in host macrophages. Iron is necessary for M. tuberculosis growth; therefore, activation of multiple virulence mechanisms to acquire iron from the host environment is an important survival strategy for M. tuberculosis in macrophages (1). The elevation of M. tuberculosis survival by exogenous iron supplementation (Supplemental Methods and Supplemental Figure 1A; supplemental material available online with this article; https://doi.org/10.1172/JCI184095DS1) reinforces the critical role of iron in mycobacterial infection in macrophages.

Intracellular iron homeostasis is achieved by regulating ferritin, an intracellular iron-storage protein comprising light (FTL1) and heavy (FTH1) chains. Corroborating the previous study (2), we found that M. tuberculosis infection induced ferritin expression (Supplemental Figure 1B), and inhibition of ferritin (Supplemental Figure 1C) resulted in an enhancement of iron availability (Supplemental Figure 1, D–F) and survival of intracellular M. tuberculosis (Supplemental Figure 1G) in macrophages, suggesting that ferritin-dependent iron restriction is a crucial immune defense mechanism against mycobacterial infection.

Dai et al. have shown that M. tuberculosis hijacks the ferritinophagy pathway to degrade ferritin, thereby increasing intracellular iron availability (2); however, ferritin breakdown is also executed by ubiquitin-conjugating enzyme E2O (UBE2O) (3). Therefore, to investigate the contribution of the UBE2O pathway in iron-based interaction between macrophages and mycobacteria, we infected macrophages with M. tuberculosis and immunoprecipitated FTH1 at different time points, followed by immunoblotting analysis. We found that mycobacterial infection triggered ferritin ubiquitination (Figure 1A) and interaction between ferritin and UBE2O (Figure 1B), implying an important role of UBE2O in ferritin regulation by M. tuberculosis.

We generated UBE2O-KO cells (Figure 1C) and analyzed ferritin expression in the context of M. tuberculosis infection. We found no difference at the basal level of ferritin in wild-type and UBE2O-KO cells; however, mycobacterial infection induced a higher ferritin level in UBE2O-KO cells compared with controls (Figure 1C). Furthermore, FTH1 immunoprecipitation, followed by immunoblot analysis, revealed that M. tuberculosis failed to trigger ferritin ubiquitination in UBE2O-KO macrophages (Figure 1D). Additionally, treatment with the proteasome inhibitor MG132 increased mycobacteria-induced ferritin levels in wild-type cells but not in UBE2O-KO cells (Supplemental Figure 2). These findings indicate that in addition to the ferritinophagy machinery (2), M. tuberculosis also hijacks the UBE2O pathway to regulate host ferritin.

Choudhary et al. reported that M. tuberculosis infection induces phosphorylation at different serine residues of UBE2O (4); therefore, we mutated these serine (S) residues to alanine (A) and analyzed their roles in ferritin regulation. All mutations did not influence the basal level of ferritin; however, mycobacterial infection induced a higher ferritin level in UBE2O-KO and S82A mutant clones but not in S269A and S893A mutant clones compared with controls (Figure 1E), suggesting that S82 phosphorylation is required for ferritin degradation induced by M. tuberculosis. The inability of S82A mutant UBE2O to interact with ferritin in the context of M. tuberculosis infection (Figure 1F) confirmed an indispensable role of S82 in UBE2O-dependent ferritin regulation.

We next evaluated the contributions of the UBE2O-dependent ferritin degradation mechanism to M. tuberculosis infection in macrophages. We found that inactivation of the UBE2O pathway reduced mycobacterial survival in macrophages; M. tuberculosis readily proliferated within control and UBE2O[S893A] mutant macrophages but failed to grow in UBE2O KO and UBE2O[S82A] mutant macrophages (Figure 1G). However, the difference in mycobacterial survival in UBE2O-KO and UBE2O[S82A] mutant macrophages at 48 hours after infection suggests that UBE2O-dependent bacterial survival is also mediated by unknown ferritin-independent mechanism(s). To analyze the role of the UBE2O pathway in the survival of mycobacteria in primary macrophages, we infected mouse alveolar macrophages with M. tuberculosis, the first lung cells to encounter M. tuberculosis, and concurrently treated them with arsenic trioxide (ATO), an inhibitor of UBE2O expression and activity (5, 6). We found that ATO at noncytotoxic concentrations inhibited UBE2O-dependent ferritin degradation (Supplemental Figure 3, A–C), thereby reducing M. tuberculosis survival in macrophages (Figure 1H). Additionally, ATO treatment markedly reduced M. tuberculosis burden in mouse lungs (Figure 1I), confirming an important role of UBE2O in M. tuberculosis infection in vivo.

In summary, our study identified a crucial virulence mechanism used by M. tuberculosis to regulate macrophage iron homeostasis. Through triggering serine 82 phosphorylation, M. tuberculosis activates UBE2O to ubiquitinate ferritin protein, leading to ferritin breakdown and iron release to the cytosol for mycobacterial growth. Furthermore, the reduction of M. tuberculosis survival in macrophages and mice by ATO-dependent UBE2O inhibition suggests that the development of arsenic-based drugs could be a promising therapeutic strategy for treating tuberculosis, particularly in individuals with immunosuppression due to cancers or cancer treatments, because ATO is a first-line treatment drug for acute promyelocytic leukemia (APL) and has also shown antitumor effects in various cancers (5).

## Supplementary Material

Supplemental data

Unedited blot and gel images

Supporting data values

## Figures and Tables

**Figure 1:**
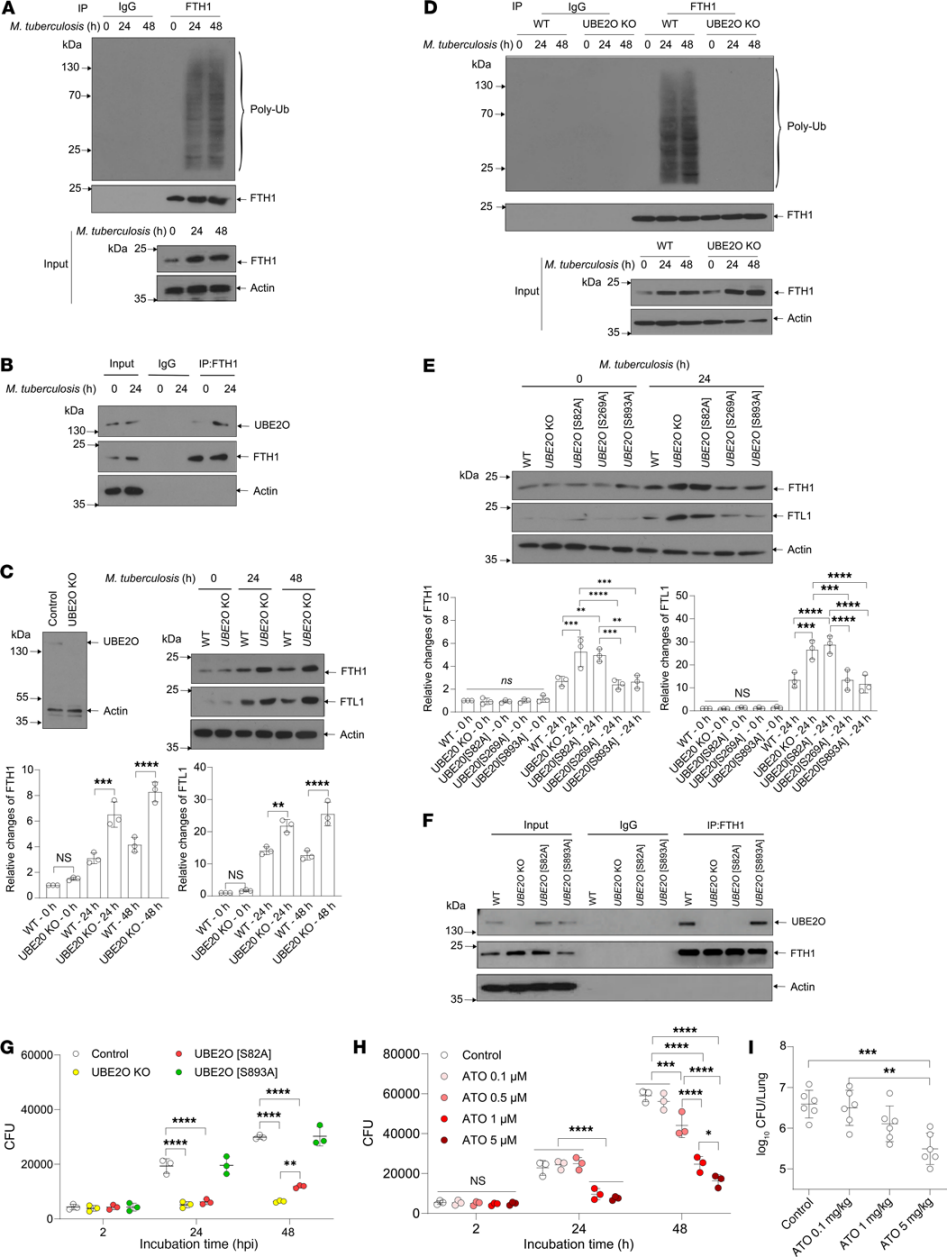
*M*. *tuberculosis* survival is mediated by UBE2O-dependent ferritin degradation. (**A**) Mycobacterial infection triggers ferritin ubiquitination in J774A.1 macrophages. (**B**) Mycobacterial infection induces ferritin-UBE2O interaction in J774A.1 cells. (**C**) UBE2O KO is validated by immunoblotting (upper left panel). Loss of UBE2O enhances *M*. *tuberculosis*-–induced ferritin expression in J774A.1 macrophages. Immunoblot analysis of UBE2O protein (upper right panel); protein level was quantified by ImageJ (NIH) and normalized to respective actin (lower panels). (**D**) Indispensable role of UBE2O in ubiquitin-dependent ferritin degradation. (**E**) Residue serine 82 is required for UBE2O-dependent ferritin degradation. Immunoblot analysis of FTH1 and FTL1 proteins (upper panel); protein level was quantified by ImageJ and normalized to respective actin (lower panels). (**F**) Mutant UBE2O at serine 82 fails to interact with ferritin. (**G**) Inactivation of UBE2O reduces mycobacterial survival in macrophages. (**H**) Treatment with arsenic trioxide, a UBE2O inhibitor, restricts mycobacterial survival in alveolar macrophages. (**I**) Inhibitory effect of ATO on *M*. *tuberculosis* infection in mice. Bacterial burden was analyzed at day 28 after infection. *P < 0.05; **P < 0.01; ***P < 0.001; ****P < 0.0001.

## Version 1 05/01/2025

Electronic publication
