# Supplementary material for: Mycobacterium tuberculosis hijacks the UBE2O pathway to regulate host iron homeostasis
Source: J Clin Invest. 2025 May 1;135(9):e184095. doi: 10.1172/JCI184095 (PMC12043076; doi:10.1172/JCI184095)
Supplement: Supplemental data [file jci-135-184095-s045.pdf]

Title: ***Mycobacterium tuberculosis* hijacks the UBE2O pathway to regulate host iron homeostasis**

Authors: Tran Xuan Ngoc Huy[1], Huynh Tan Hop[2]*

Affiliations

[1]Institute of Animal Medicine, Gyeongsang National University, Jinju, Republic of Korea, [2]University Center for Bioscience and Biotechnology, National Cheng Kung University, Tainan, Taiwan

* Correspondence to: z11005045@ncku.edu.tw

MATERIALS AND METHODS

Sex as a biological variable

Sex was not considered as a biological variable.

Reagents

Antibodies and reagents used in this study were from the following sources: rabbit monoclonal anti-FTH1 antibody (D1D4) (#4393T), mouse monoclonal anti-β-actin antibody (#3700S), and MG132 (#2194) from Cell Signaling; BD cytofix/cytoperm (#BD554714), live/dead fixable near-IR dead cell stain kit (#L10119), mouse monoclonal anti-FTH1 antibody (18C10) (#MA5-50221), rabbit polyclonal anti-FTL1 antibody (#PA-27357), mouse monoclonal anti-ubiquitin antibody (eBioP4D1 (P4D1)), eBioscience (#14-6078-82), rabbit polyclonal anti-UBE2O antibody (#PA5-87583), goat anti-mouse IgG-HRP (#31430), goat anti-rabbit IgG-HRP (#31460), rabbit IgG isotype control (#02-6102), Lipofectamine RNAiMAX (#13778030), FBS (#26140079), 7H9 media (#DF0713-17-9), OADC enrichment (#B12351), BCA protein assay kits (#23225), and halt protease and phosphatase inhibitor cocktail (#1861284) from Thermo Fisher Scientific; Calcein AM (#17783) and Deferoxamine mesylate salt (DFO, #D9533) from Sigma; control siRNA (#sc-37007) and Fth1 siRNA (#sc-40576) from Santa Cruz; DMEM high glucose (#11965092) and penicillin/streptomycin (#15140122) from Gibco.

Bacterial and cell culture

M. tuberculosis H37Rv (ATCC, #25618) was cultured in 7H9 media supplemented with 10% OADC enrichment at 37 ^{o}C in a biosafety laboratory level 3 containment facility until the stationary phase.

Mouse macrophages (J774A.1, ATCC, #TIB-67) were grown in DMEM high glucose supplemented with 10% FBS and 1% penicillin/streptomycin. Cells were incubated at 37 ^{o}C and 5% CO2.

Infection assay

M. tuberculosis survival in macrophages was evaluated by colony-forming unit (CFU) assay as described before (1). In brief, macrophages were infected with *M. tuberculosis* at MOI of 10 for 1 hour, followed by elimination of extracellular bacteria with medium containing Amikacin (200 μg/ml) for 2 hours, and re-cultured in antibiotic-free culture medium until indicated time points. Cells were washed three times with PBS, lysed in ddH_2O for 10 min, serially diluted in PBS, and plated on 7H10 agar plates. Bacterial CFU was determined after 3-4 weeks of incubation at 37 ^{o}C.

Ferric ammonium citrate (FAC) (10 and 100 μM) was concurrently treated with infection for iron supplementation assay.

Alveolar macrophages (AMs) were isolated from mouse lungs as previously described (2) and

cultured in DMEM supplemented with 10% FBS, 20% L-929 culture supernatant, 1 mM Sodium Pyruvate, 10 mM HEPES, and 1% penicillin/streptomycin. Cells were infected with *M. tuberculosis* and concurrently treated with arsenic trioxide (ATO) at 0.1, 0.5, 1, and 5 μM concentrations. The bacterial burden was determined at the indicated time points using the CFU method described above.

Intracellular labile iron quantification and bacterial survival analysis in Fth1-suppressing macrophages

Cells were transfected with control or Fth1 siRNAs using Lipofectamine RNAiMAX for 12 h and infected with *M. tuberculosis*. Bacterial survival at different time points was determined by CFU assay as described above in the infection assay.

At 24 h post-infection, the labile iron pool was determined using the Calcein-AM as previously described (3, 4). Briefly, cells were washed with PBS and treated with Calcein-AM (0.5 μM) for 30 min at 37 ^{o}C, 5% CO_2. Cells were incubated with Live/Death for 30 min at 4°C, followed by fixation and permeabilization with cytofix/cytoperm at room temperature for 1 hour. Cells were washed, harvested, resuspended in 200 μl FACS buffer (PBS, 2% FCS, 0.1% NaN3, 5 mM EDTA), and analyzed by the BD FACS CantoII Analyzer. The results were analyzed with FlowJo software (Tree Star). The percentage of labile iron is inversely proportional to the fluorescence of Calcein-AM. Positive and negative controls are cells treated with $FeSO_4$ (10 μM) and DFO (10 μM), respectively.

Cell lysis and immunoblotting

Cells were lysed in RIPA buffer (50 mM Tris-HCl pH7.4, 150 mM NaCl, 1% NP-40, 0.5% Na deoxycholate (NaDOC), 0.1% SDS, 1mM EDTA) containing protease and phosphatase inhibitor cocktail for 30 min at 4°C. Protein lysate was quantified by BCA assay, separated on 10 or 12% SDS-PAGE gels, and transferred onto nitrocellulose membranes with wet transfer systems (BioRad, USA). The membranes were blocked with 5% BSA in PBS-Tween20 for 1 hour at room temperature, followed by primary antibody incubation overnight at 4 ^{o}C. The primary antibodies were diluted in 5% BSA in PBS-Tween20 at 1:2,000 for anti-actin antibody and 1:1,000 for other antibodies. The membranes were washed, incubated with horseradish peroxidase (HRP)-conjugated secondary antibodies for 1 hour at room temperature, washed, developed by WesternBright quantum HRP substrate, and imaged using film processor.

Co-immunoprecipitation and immunoblotting

Co-immunoprecipitation was performed as previously described (5-7). Briefly, cells were washed and lysed in NETN buffer (150 mM NaCl, 20 mM Tris-HCl (pH 7.5), 1 mM EDTA, 0.5% NP-40, and protease and phosphatase inhibitor cocktail). The lysates were incubated with magnetic beads cross-linked with control IgG or anti-FTH1 antibodies (2 μg antibodies for each 200 μg lysate sample) at 4 °C overnight. After magnetic separation, precipitates were washed 3 times with NETN buffer and quantified by BCA assay. The samples were analyzed by immunoblotting as described in the "Cell lysis and immunoblotting" section above.

Generation of UBE2O knockout or mutant macrophages by CRISPR/Cas9 knock-in

Generation of UBE2O knockout or mutant J774A.1 macrophages using the homologous directed repair (HDR)-based knock-in was performed as previously described (4). In brief, 2 nmol crRNA (KO 5'-GAC TGT GCC GTC AAG CTC AT-3'; S82A 5'-CCT CAT CCA CGG CGA GGA CT-3'; S269A 5'-GCC AAG ATC TTC TCC AGT GT-3'; and S893A 5'-GGG CCA CTC GGC TTT CAC TG-3') synthesized by IDT were incubated with 5 nmol tracrRNA (IDT, #1073189) at 95 °C for 5 min. For each transfection, 0.22 nmol gRNA complex was mixed with 0.18 nmol Crispr-Cas9 protein (IDT, #1081060) and 0.2 µl of buffer R (Thermo Fisher, #MPK10096), followed by room temperature incubation for 30 min to form RNP complex. The RNP complex was mixed with 0.5 µl of 12 µM ssODN templates synthesized by Bioneer (KO 5'- TTC CTC CTC TAG GAC AGC CAG TGT GGC ACG GTG ATA GAT GTC AAC ATT GAC TGT GCC GTC TAG CTC ATC GGC ACC AAC TGC ATC ATC TAC CCT GTC AAC AGC AAG GAC CTC CAG CAC ATC TGG -3'; S82A 5'-TCC GGC CGT TAC CGC GGC TCG GTG CAC TTC GGG CTG GTA CGC CTC ATC CAC GGC GAG GAC GCG GAC TCG GAG GGC GAC GAT GAC GGC CGC GGC AGC TCG GGC TGC TCC GAG GCC GGG GGC GCG-3'; S269A 5'-TTT GAT GAC TCT TAT GGC TTC TAC CCA GGC CAA GTC CTT ATT GGC CCA GCC AAG ATC TTC GCC AGT GTC CAG TGG CTC TCT GGG GTC AAG CCT GTA CTC AGC ACC AAG AGC AAG TTC CGT GTG-3'; and S893A 5'-GAG GAG AAG ATG GAG GCA GTA CCC GAC ACA GAG CGC AAG GAG GAG AAG CCT GAG GTG CAG GCC CCA GTG AAA GCC GAG TGG CCC AGT GAG ACA CCT GTG CTC TGT CAG CAG TGC GGT GGC AGG-3', underline indicates the substitute codon), and 0.5 µl of 43.2 µM electroporation enhancer (IDT, #1075915). The 10 µl of the mixture was electroporated to 2 x 10^5 cells in antibiotic-free culture medium using 1720 V, 10 ms, 3 pulses program of Neon transfection system. The cells were transferred to antibiotic-free culture medium in 24 well culture plates for 5 days and subjected to single-cell screening. The genetic modifications were then validated by T7 endonuclease assay, DNA sequencing, and immunoblotting.

In vivo M. tuberculosis infection

Twenty-four male C57BL/6J mice, aged 8 weeks, were purchased from Jackson Laboratories and randomly allocated into four groups (six mice per group). Each mouse was aerosolized with approximately 1,000 CFU of *M. tuberculosis* using the Glas-Col inhalation system and daily received i.p. injections of PBS (control group) or ATO at doses of 0.1, 1, and 5 mg/kg in 100 µl. On day 28 after infection, mice were sacrificed, and lungs were isolated and homogenized in sterile PBS. Serial dilutions of each organ were then plated on 7H10 agar. Bacterial CFU was determined after 3-4 weeks of incubation at 37 °C.

Statistical analyses

Statistical analyses were performed with one-way or two-way ANOVA followed by multiple comparisons among groups in Prism (GraphPad Software). The results are mean values ± standard deviations from three independent experiments unless otherwise defined in the legends. Error bars show standard deviation. *, $p<0.05$; **, $p<0.01$; ***, $p<0.001$; ****, $p<0.0001$; ns, not significant.

Study approval

The animal experiment in this study was approved by the Animal Ethical Committee of Gyeongsang National University in Jinju City, Republic of Korea (authorization GNU-170331-M0017).

Data availability

Supporting data values for all graphs can be found in an accompanying XLS file.

Acknowledgments

This project was supported by Distinguished Scholar Program Grant No. D110-G9603 through the National Cheng Kung University, Taiwan (to HTH) and Young Researcher Program Grant No. 2018R1D1A1B07048220 (to HTH) through the National Research Foundation of Korea. We thank Chih-Hui Wu at National Cheng Kung University, Taiwan, for excellent technical assistance.

Author contributions

Conceptualization: HTH; Methodology: HTH, TXNH; Investigation: TXNH, HTH; Funding acquisition: HTH; Manuscript writing: TXNH; Manuscript review: HTH.

REFERENCES

1. Walburger A, Koul A, Ferrari G, Nguyen L, Prescianotto-Baschong C, Huygen K, et al. Protein kinase G from pathogenic mycobacteria promotes survival within macrophages. *Science.* 2004;304(5678):1800-4.
2. Busch CJ, Favret J, Geirsdottir L, Molawi K, and Sieweke MH. Isolation and Long-term Cultivation of Mouse Alveolar Macrophages. *Bio Protoc.* 2019;9(14).
3. Staubli A, and Boelsterli UA. The labile iron pool in hepatocytes: prooxidant-induced increase in free iron precedes oxidative cell injury. *Am J Physiol.* 1998;274(6):G1031-7.
4. Hop HT, Huy TXN, Lee HJ, and Kim S. Intracellular growth of Brucella is mediated by Dps-dependent activation of ferritinophagy. *EMBO Rep.* 2023;24(9):e55376.
5. Yang Q, Liao M, Wang W, Zhang M, Chen Q, Guo J, et al. CD157 Confers Host Resistance to Mycobacterium tuberculosis via TLR2-CD157-PKCzeta-Induced Reactive Oxygen Species Production. *mBio.* 2019;10(4).
6. Liu J, Ren Z, Yang L, Zhu L, Li Y, Bie C, et al. The NSUN5-FTH1/FTL pathway mediates ferroptosis in bone marrow-derived mesenchymal stem cells. *Cell Death Discov.* 2022;8(1):99.
7. Dai Y, Zhu C, Xiao W, Huang K, Wang X, Shi C, et al. Mycobacterium tuberculosis hijacks host TRIM21- and NCOA4-dependent ferritinophagy to enhance intracellular growth. *J Clin Invest.* 2023;133(8).

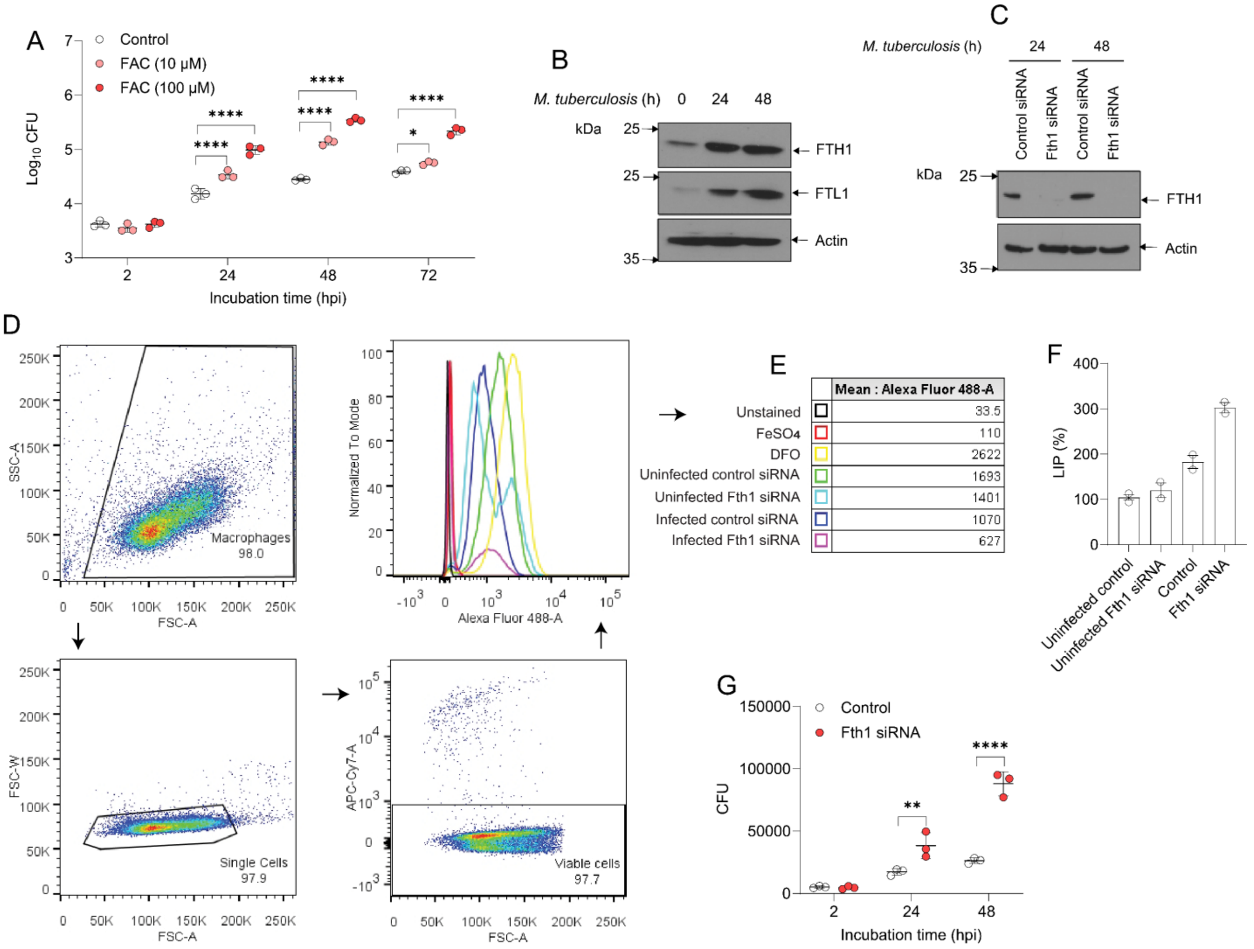

Figure S1: Ferritin induction decreases *M. tuberculosis* survival in J774A.1 macrophages by limiting intracellular iron availability

(A) Iron supplementation promotes mycobacterial survival in macrophages. Cells were infected with *M. tuberculosis* for 1 hour, followed by chase for the indicated times before lysis and determination of CFU. Ferric ammonium citrate (FAC) was concurrently treated with infection at 10 and 100 μM concentrations. (B) Mycobacterial infection induces ferritin expression in macrophages. Cells were infected with *M. tuberculosis* for 1 hour, followed by chase for the indicated times. Total proteins were isolated and analyzed by immunoblotting using antibodies against FTH1 and actin. (C) Fth1 knockdown efficiency validated by immunoblotting. Macrophages were treated with control or Fth1 siRNAs for 12 h and infected with *M. tuberculosis*. At indicated time points, total proteins were isolated and analyzed by immunoblotting using antibodies against ferritin and actin. (D) Labile iron pool (LIP) in macrophages analyzed by FACS assay. Cells were treated with control or Fth1 siRNAs for 12 h and infected with *M. tuberculosis*. At 24 h post-infection, cells were stained with calcein AM and analyzed by flow cytometry. (E) Mean floursence intensity (MFI) of calcein AM analyzed by flow cytometry in Fig. S1D. (F) Labile iron pool (LIP) ratio was calculated as the MFI value of the uninfected control divided by the MFI values of indicated conditions. (G) Suppression of Fth1 enhances mycobacterial survival in macrophages. Cells were treated with control or Fth1 siRNAs for 12 h and infected with *M. tuberculosis*. Bacterial survival was determined by the

CFU method at indicated time points.

Data information: Statistical analyses were performed with two-way ANOVA (Fig. S1, A and G) or one-way ANOVA (Fig. S1F) followed by multiple group comparisons. The results are the mean values ± standard deviations of three independent experiments, each with three technical replicates (Fig. S1, A and G) or two independent experiments, each with two technical replicates (Fig. S1F). *, $p<0.05$; **, $p<0.01$; ***, $p<0.001$; ****, $p<0.0001$; ns, not significant.

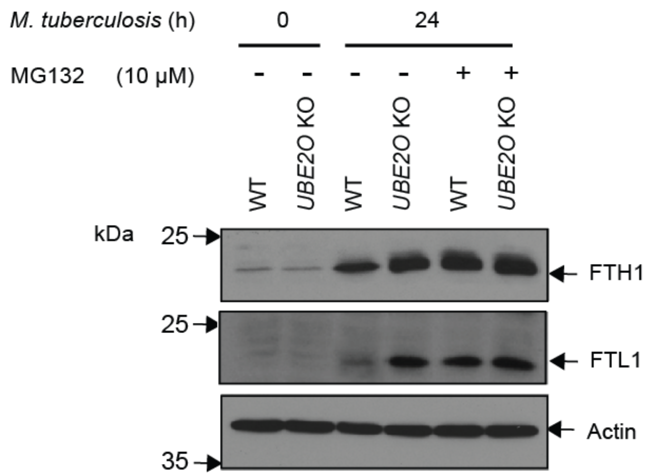

Figure S2: Proteasome inhibition abolishes UBE2O dependent-ferritin degradation in *M. tuberculosis*-infected macrophages

Wild-type and UBE2O KO macrophages were infected with *M. tuberculosis* for 1 h, followed by an 18 h chase. Cells were treated with or without 10 µM MG132, a proteasome inhibitor for 6 h and subjected to immunoblot analysis using antibodies against FTH1, FTL1 and actin.

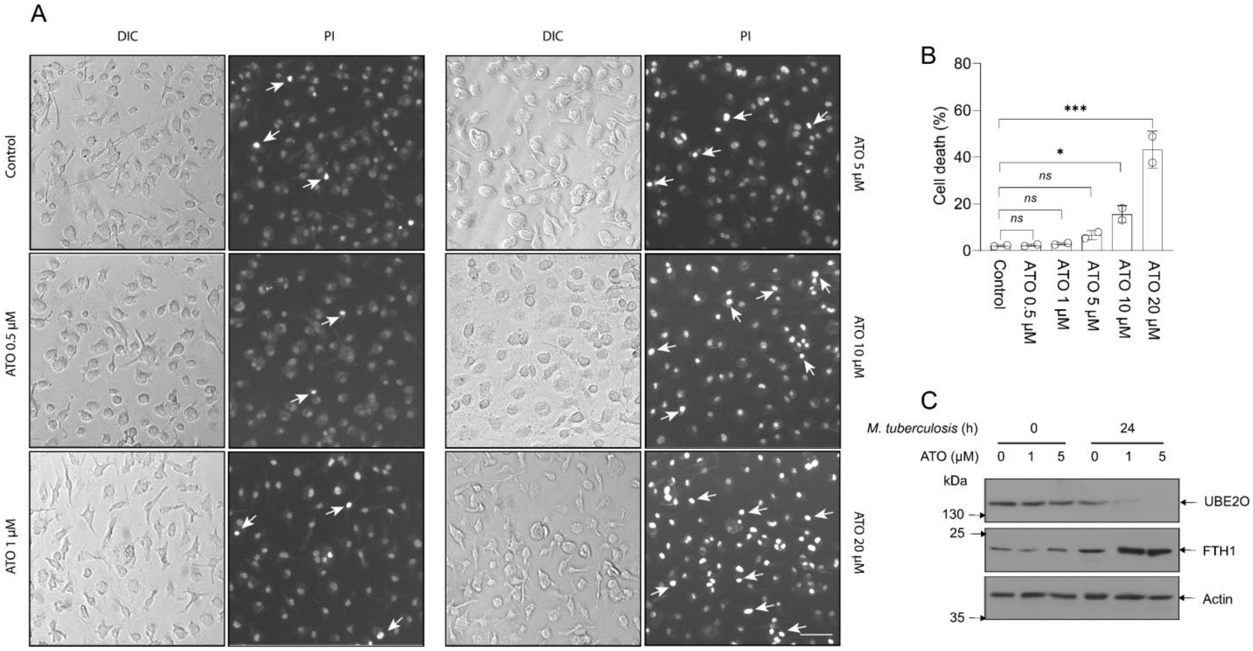

Figure S3: Inhibition of UBE2O by ATO induces ferritin expression in alveolar macrophages

(A) Alveolar macrophages were isolated from mouse lungs and treated with arsenic trioxide (ATO) at different concentrations for 48 h. Cells were stained with propidium iodide (PI) at a concentration of 1 μg/ml for 10 min and analyzed by fluorescence microscopy. (B) Quantification of cytotoxicity assay in Fig. S3A. (C) Alveolar macrophages were isolated from mouse lungs and infected with *M. tuberculosis.* Arsenic trioxide was concurrently treated with infection at 1 and 5 μM concentrations. At indicated time points, total proteins were isolated and analyzed by immunoblotting using antibodies against UBE2O, FTH1 and actin.

Data information: Statistical analyses in Fig. S3B was performed with one-way ANOVA followed by multiple group comparisons. The results are the mean values ± standard deviations of two independent experiments. *, $p<0.05$; **, $p<0.01$; ***, $p<0.001$; ****, $p<0.0001$; ns, not significant.
