# Supplementary material for: Mycobacterium tuberculosis hijacks the UBE2O pathway to regulate host iron homeostasis
Source: J Clin Invest. 2025 May 1;135(9):e184095. doi: 10.1172/JCI184095 (PMC12043076; doi:10.1172/JCI184095)
Supplement: Unedited blot and gel images [file jci-135-184095-s046.pdf]

Full unedited gel for Figure 1A

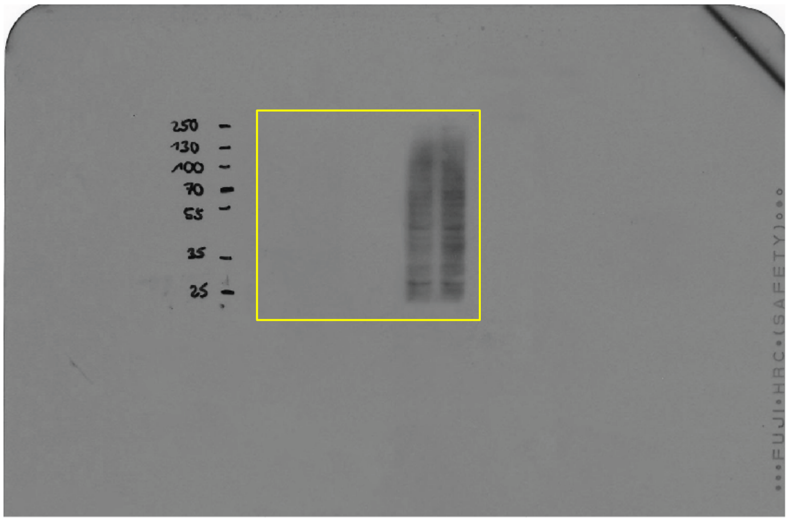

Anti-ubiquitin

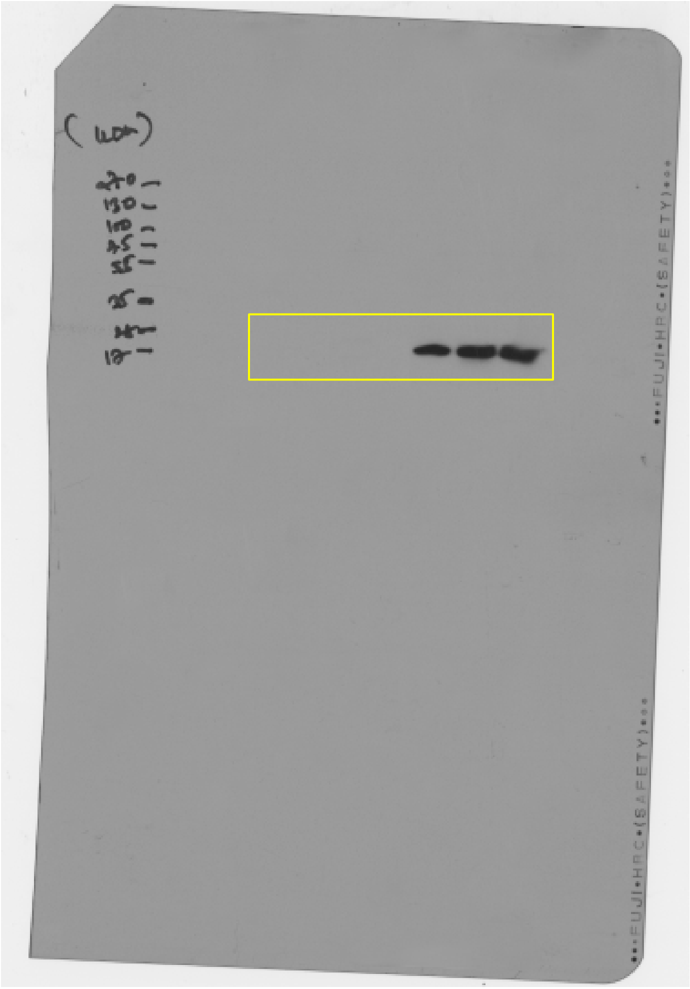

Anti-FTH1

Input

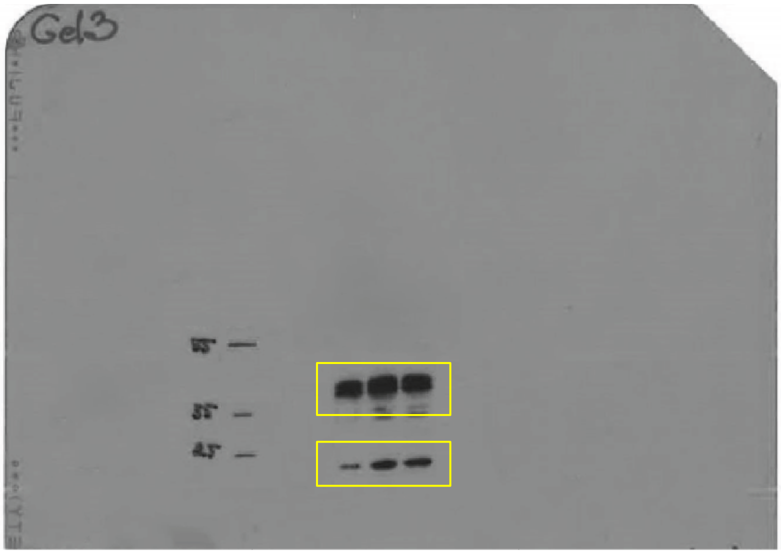

Anti-actin (top), anti-FTH1 (bottom)

Full unedited gel for Figure 1B

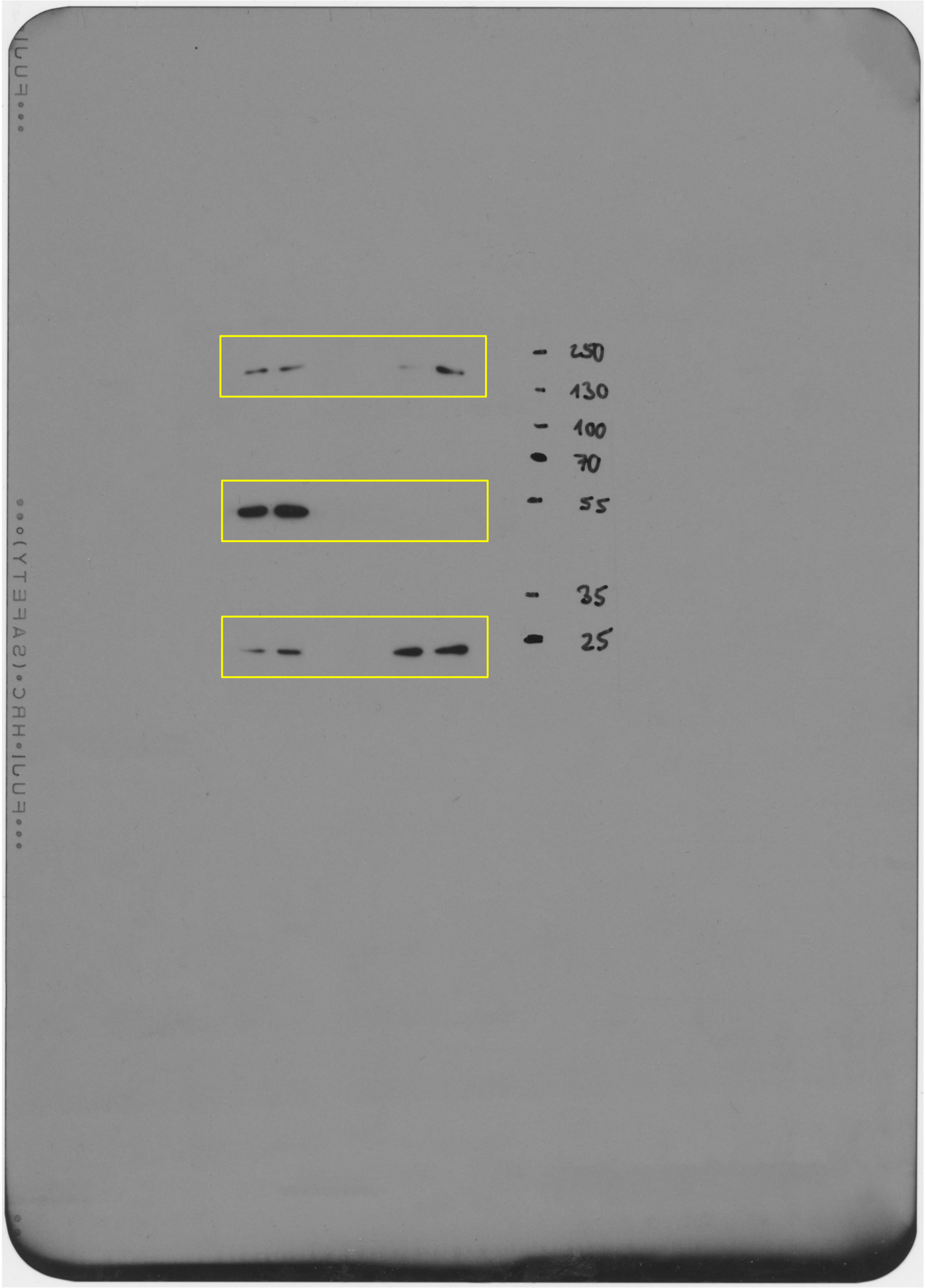

Anti-UBE2O (top), anti-actin (middle), and anti-FTH1 (bottom)

Full unedited gel for Figure 1C - left panel

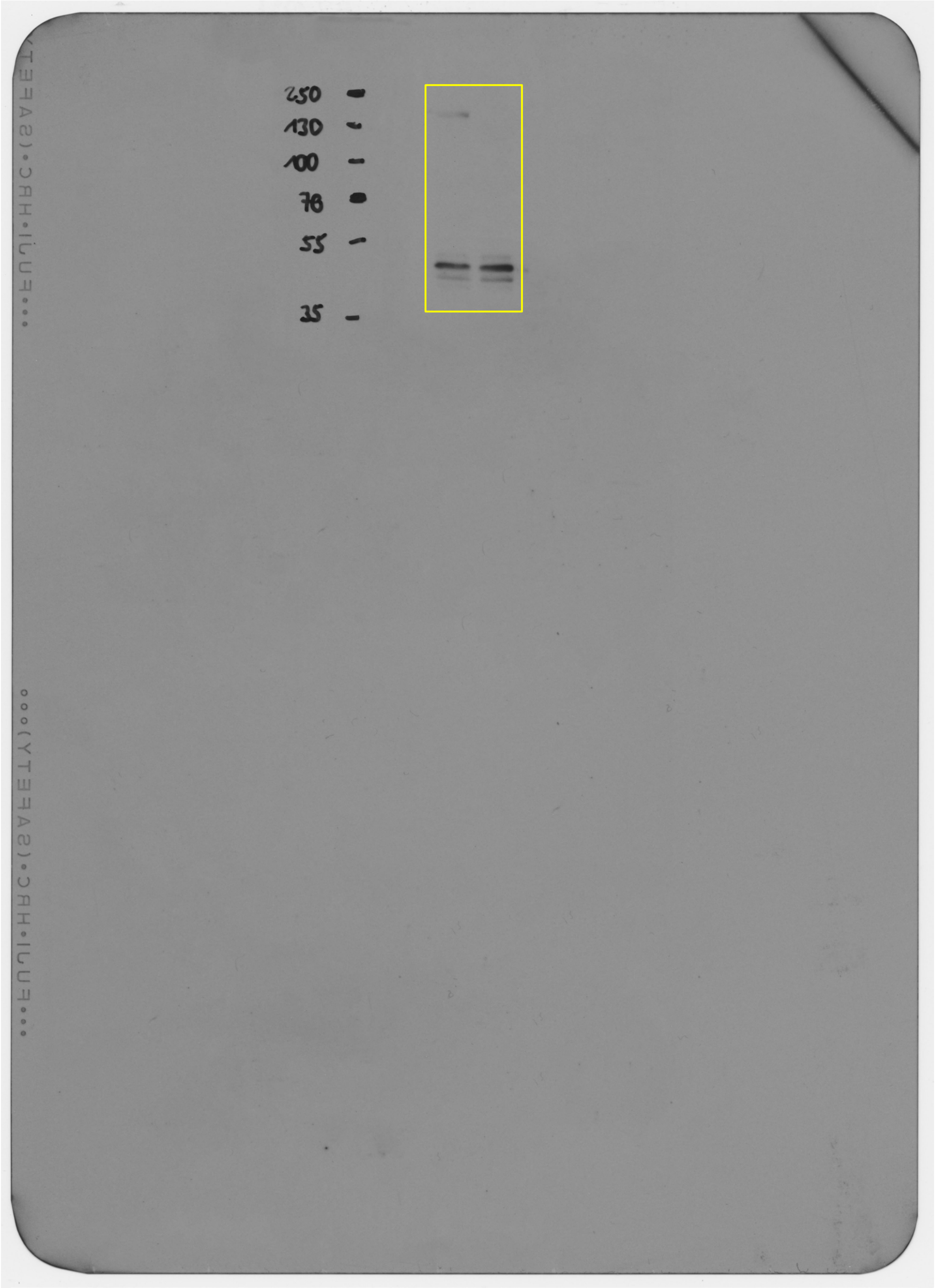

Anti-UBE2O (top) and anti-actin (bottom)

Full unedited gel for Figure 1C – right panel

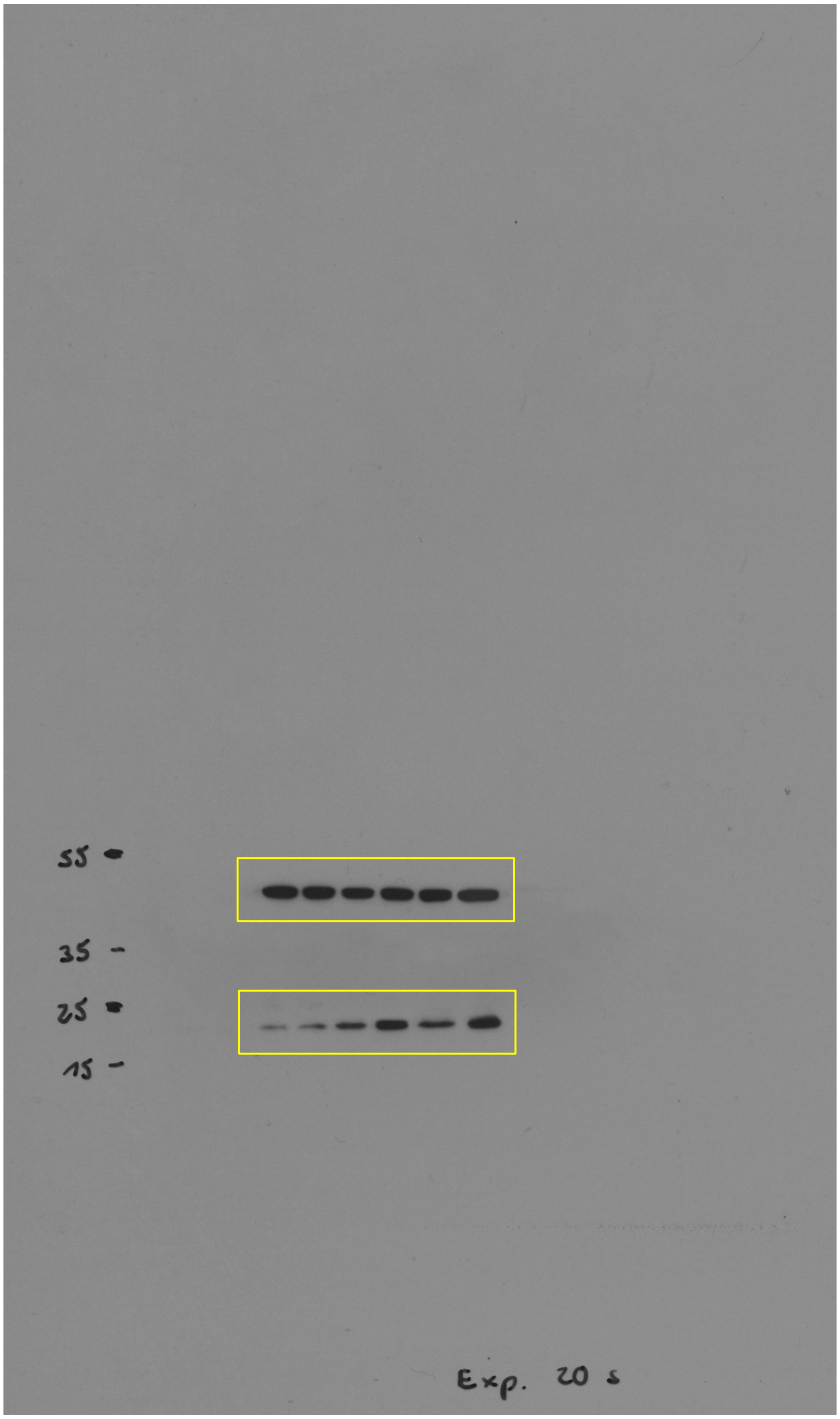

Anti-actin (top) and anti-FTH1 (bottom)

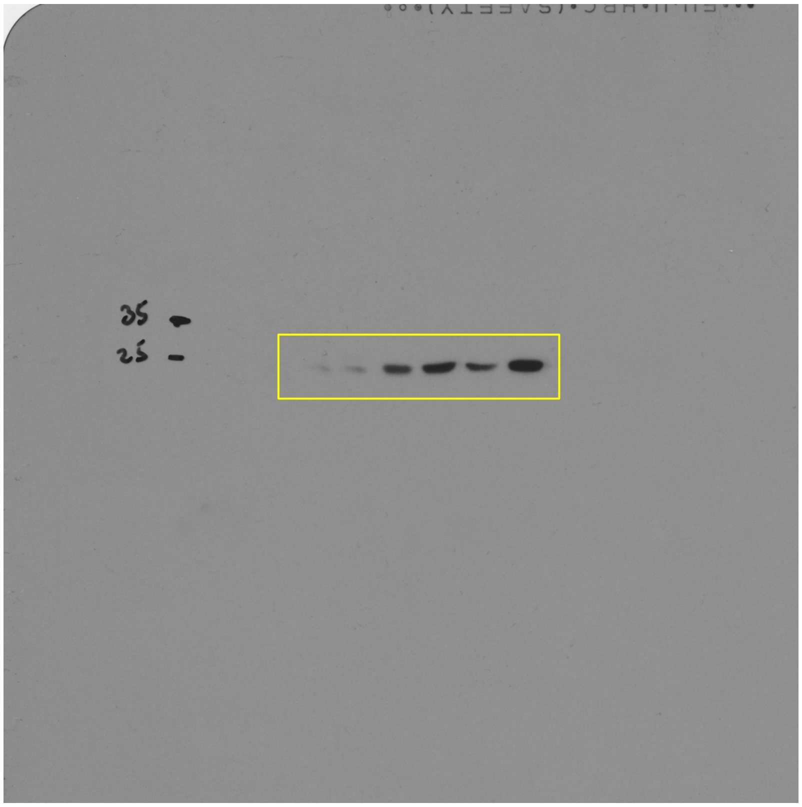

Anti-FTL1

Full unedited gel for Figure 1D

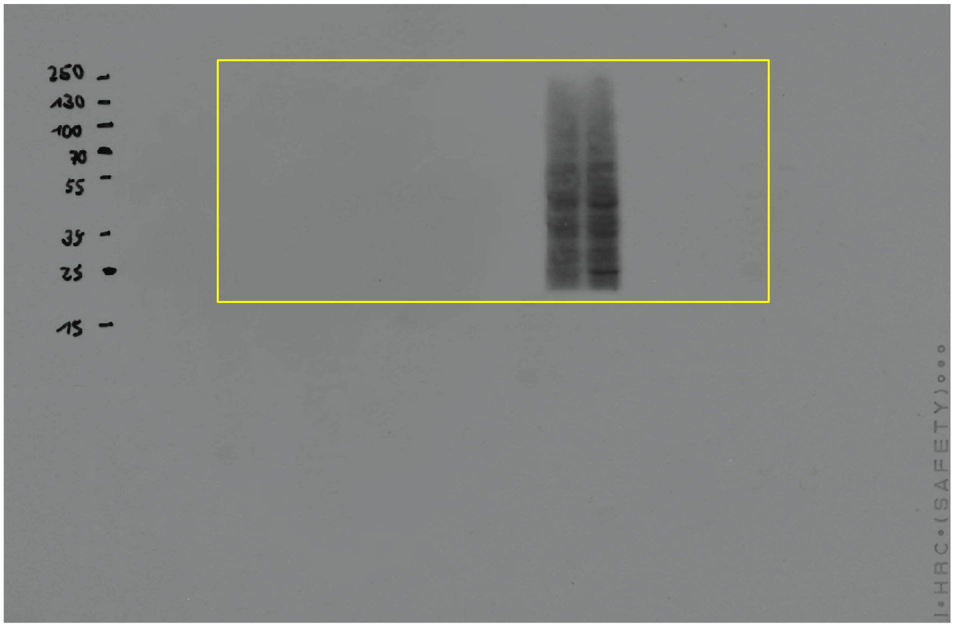

Anti-ubiquitin

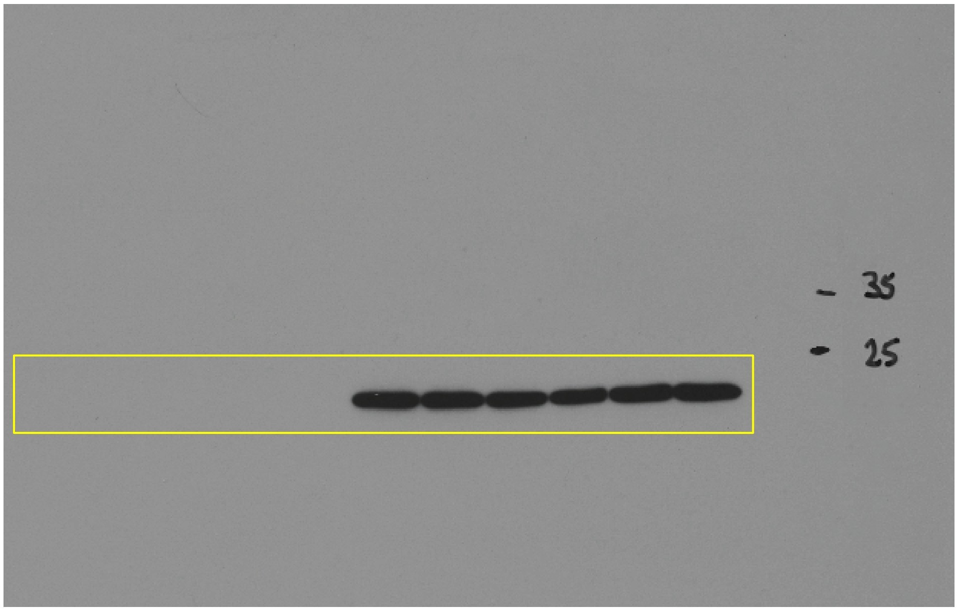

Anti-FTH1

Full unedited gel for Figure 1D - input

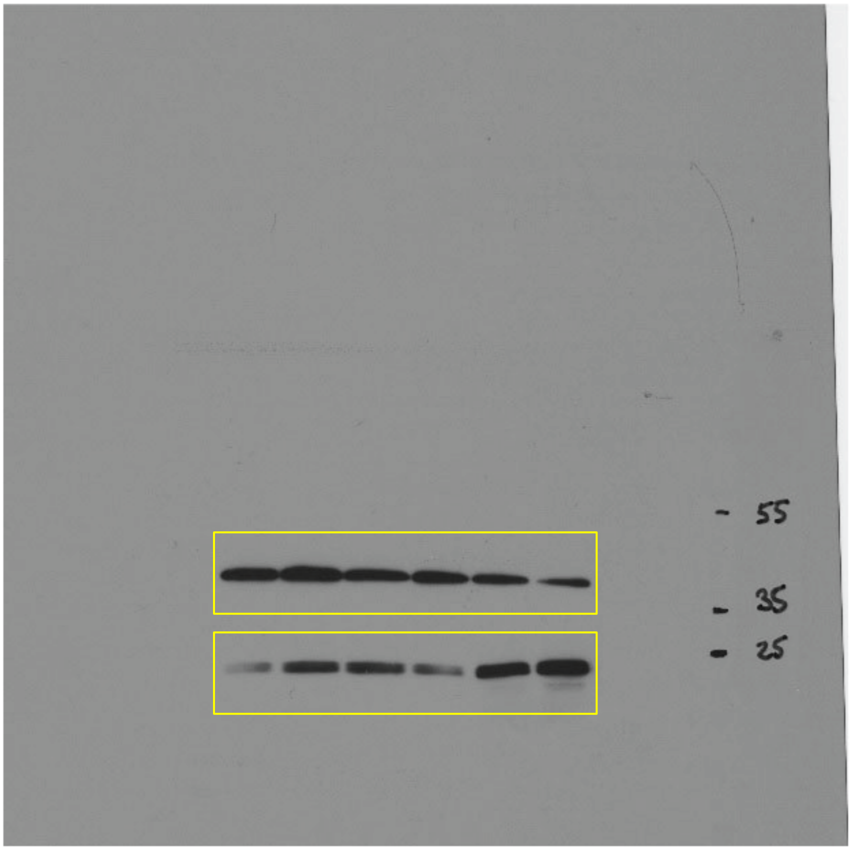

Anti-actin (top) and anti-FTH1 (bottom)

Full unedited gel for Figure 1E

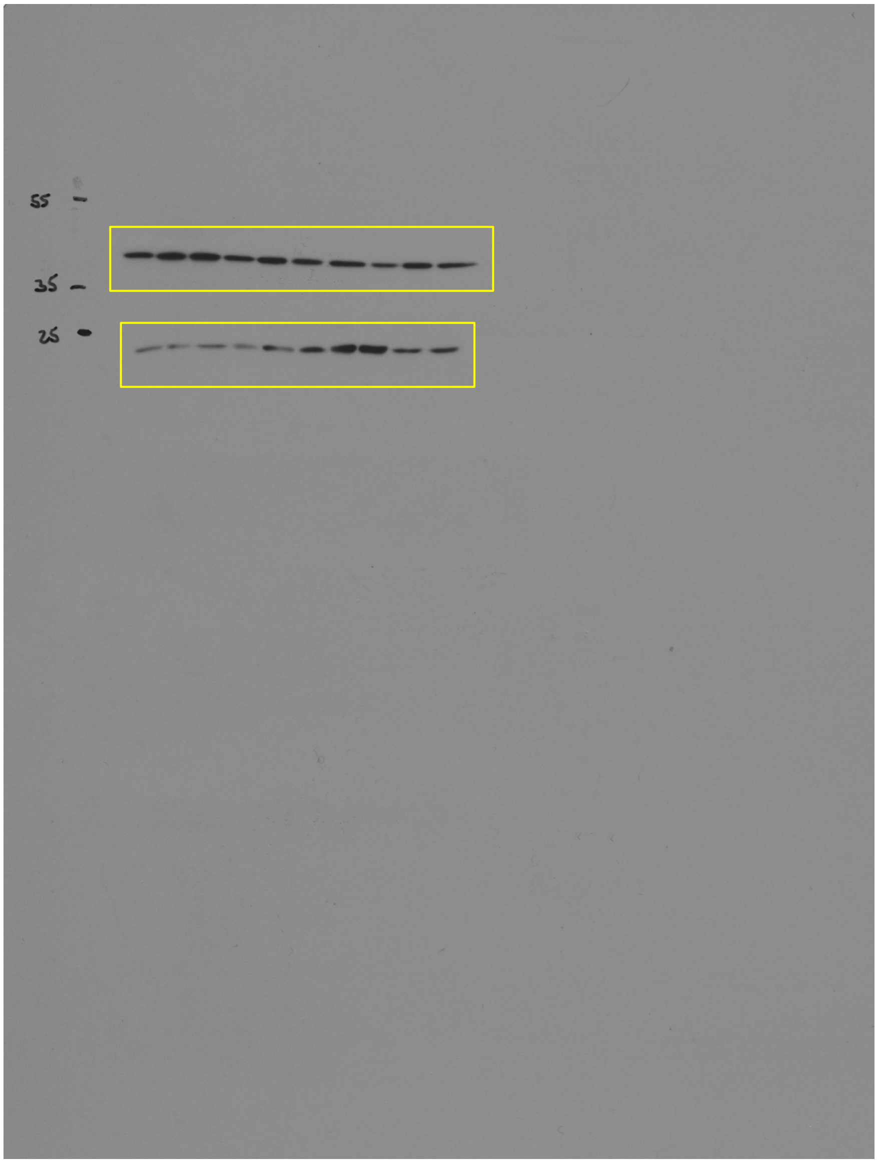

Anti-actin (top) and anti-FTH1 (bottom)

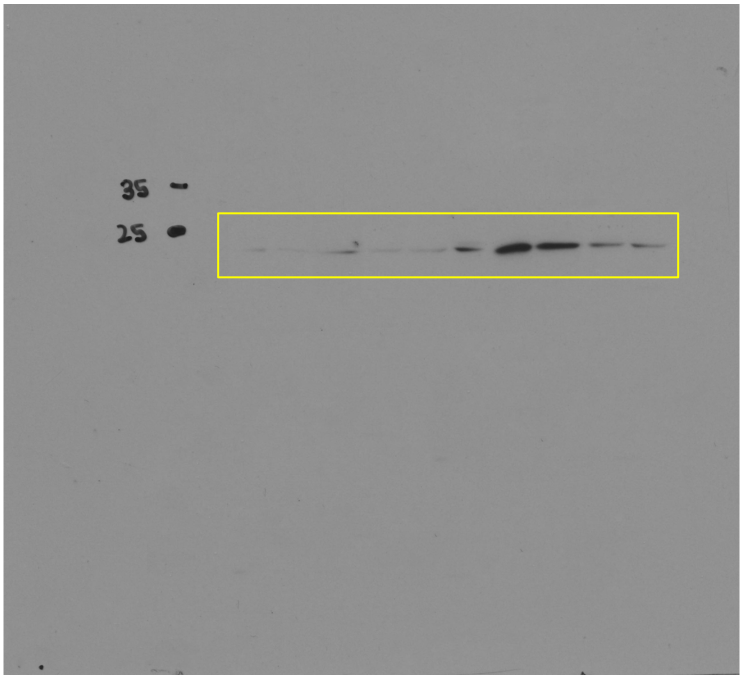

Anti-FTL1

Full unedited gel for Figure 1F

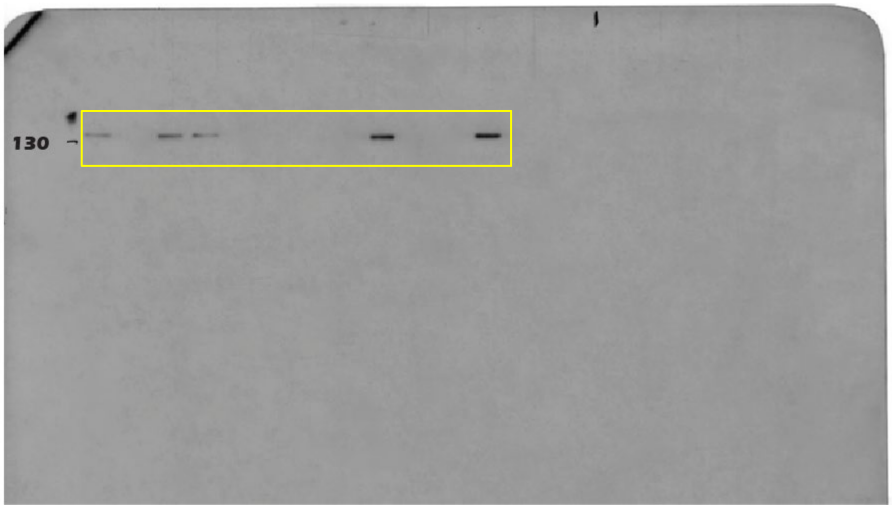

Anti-UBE2O

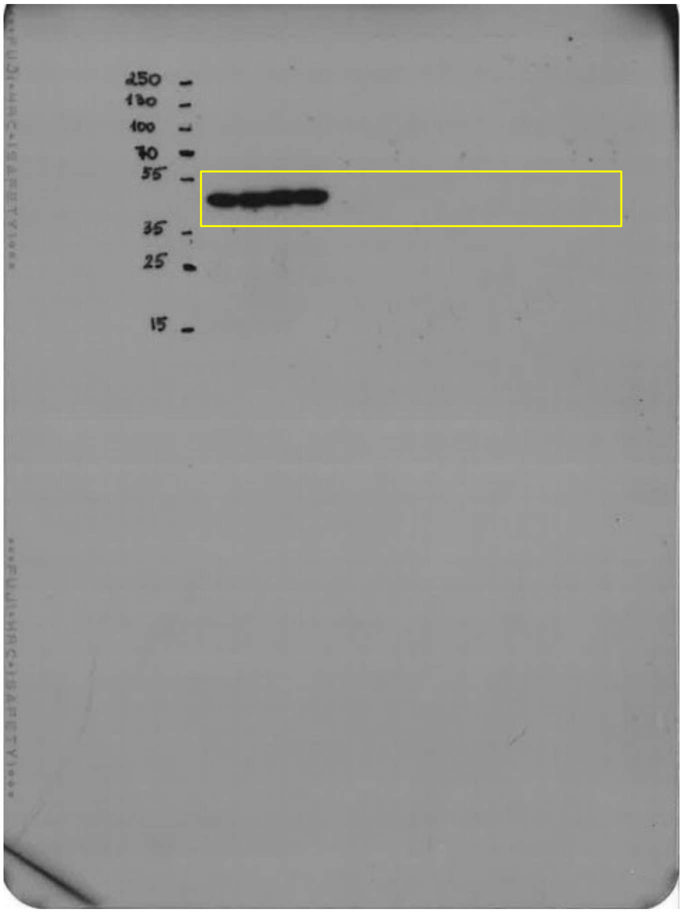

Anti-actin

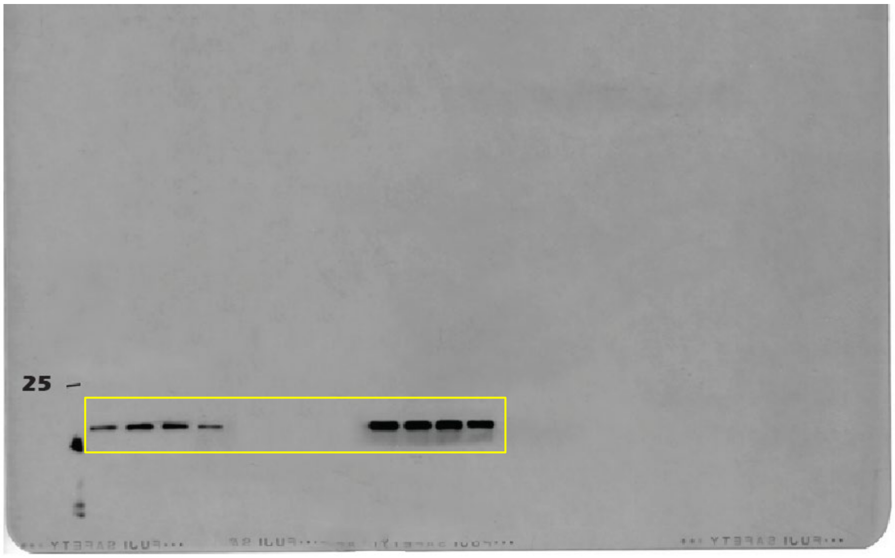

Anti-FTH1

Full unedited gel for Figure S1B

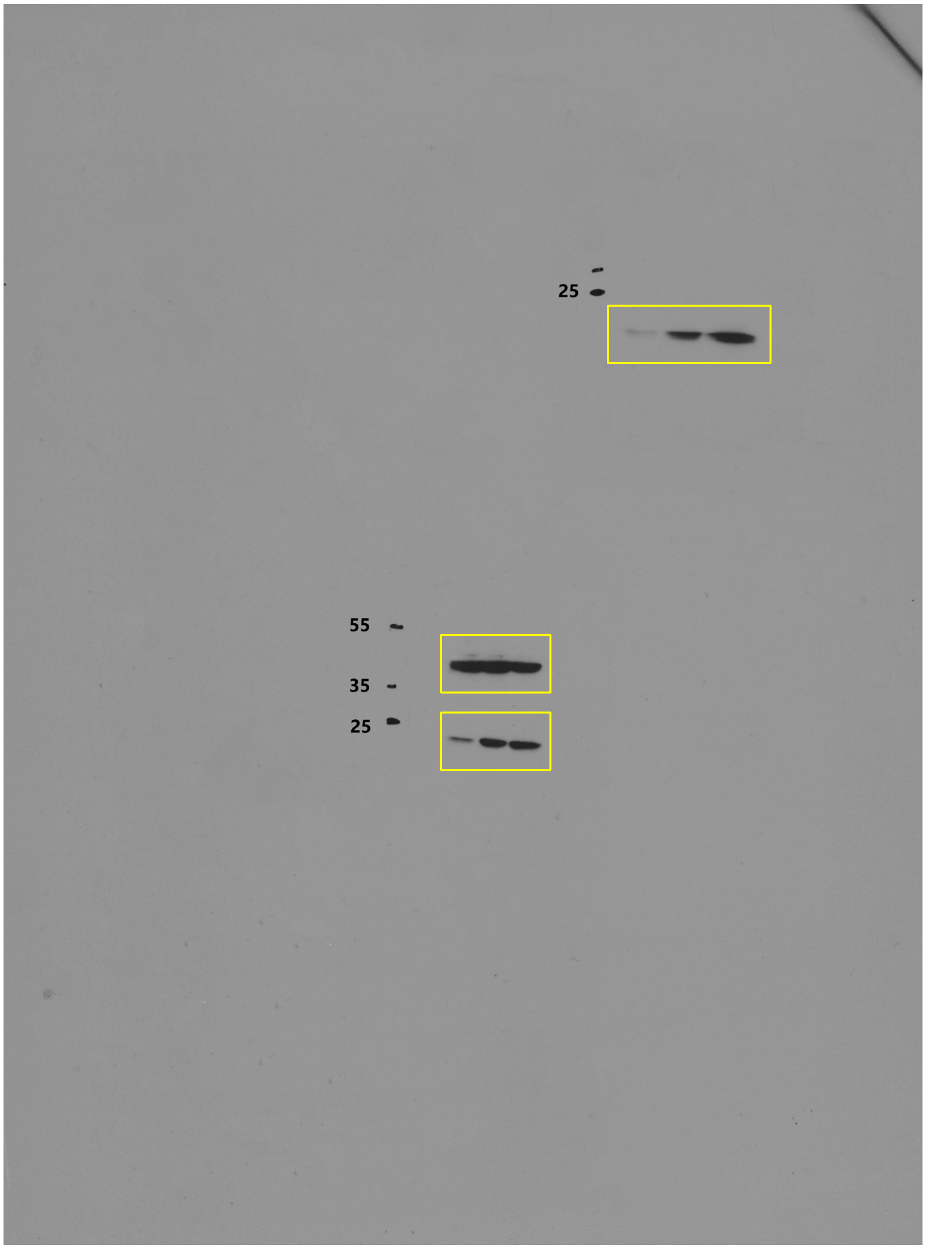

Anti-FTL1 (top), anti-actin (middle), and anti-FTH1 (bottom)

Full unedited gel for Figure S1C

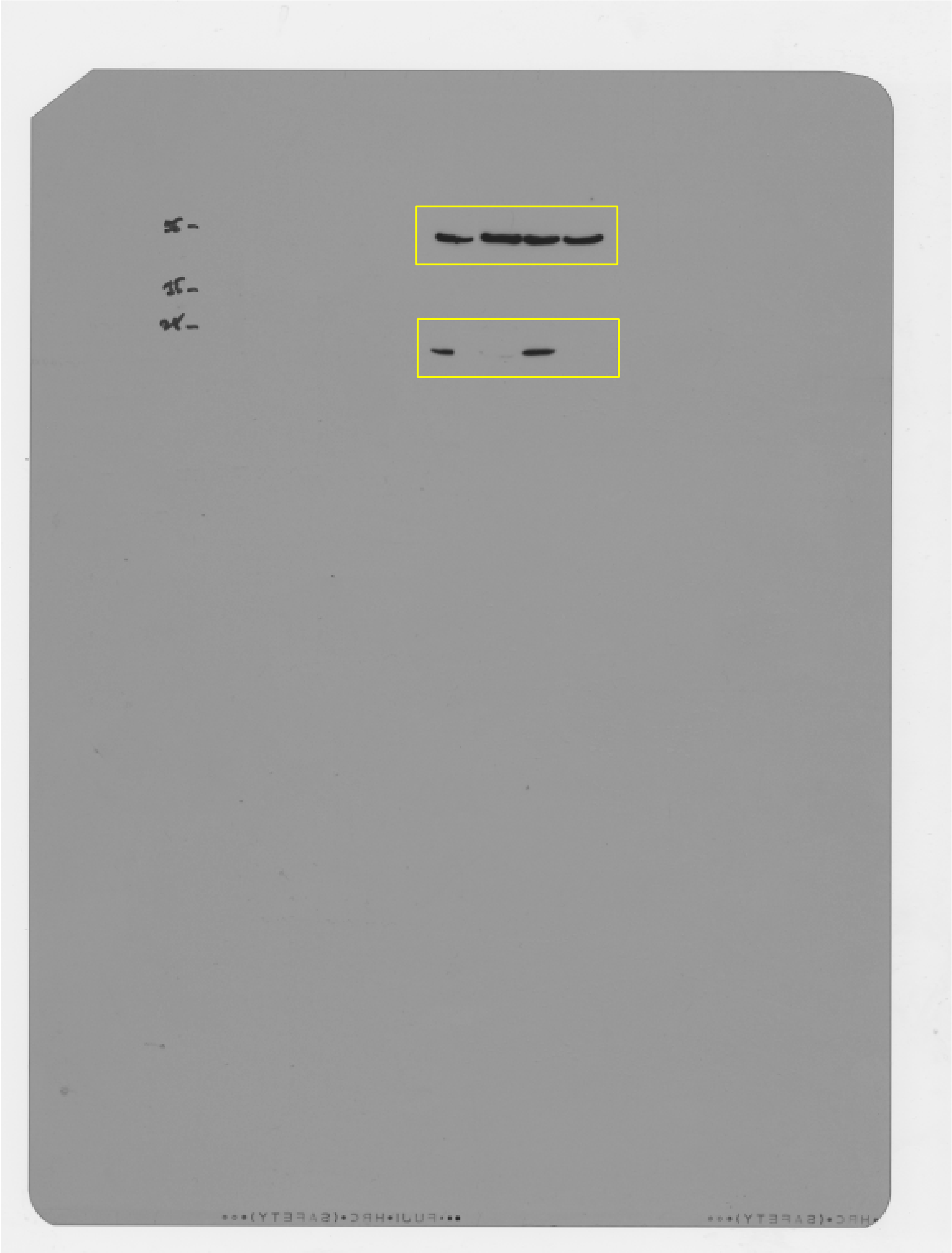

Anti-actin (top) and anti-FTH1 (bottom)

Full unedited gel for Figure S2

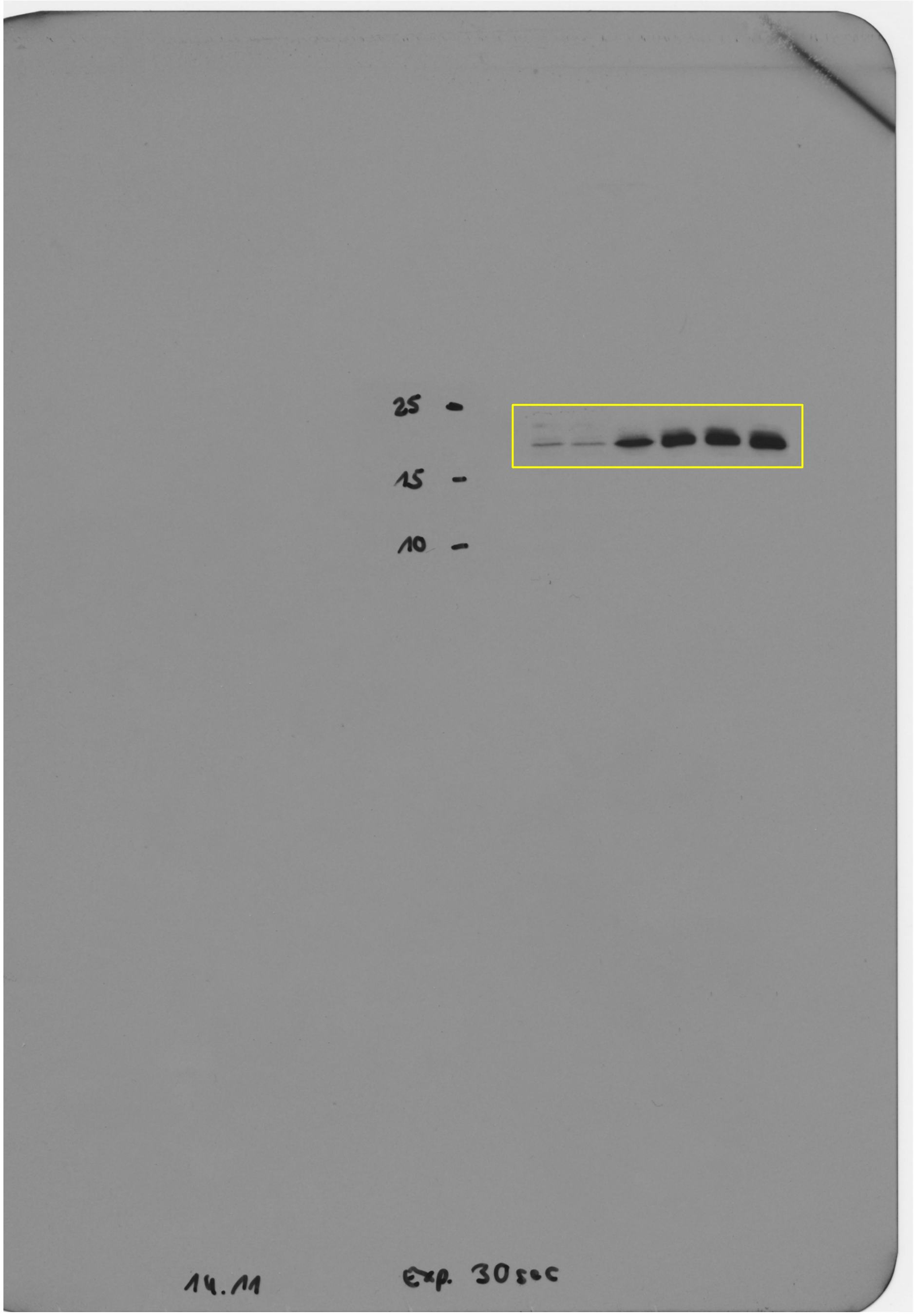

Anti-FTH1

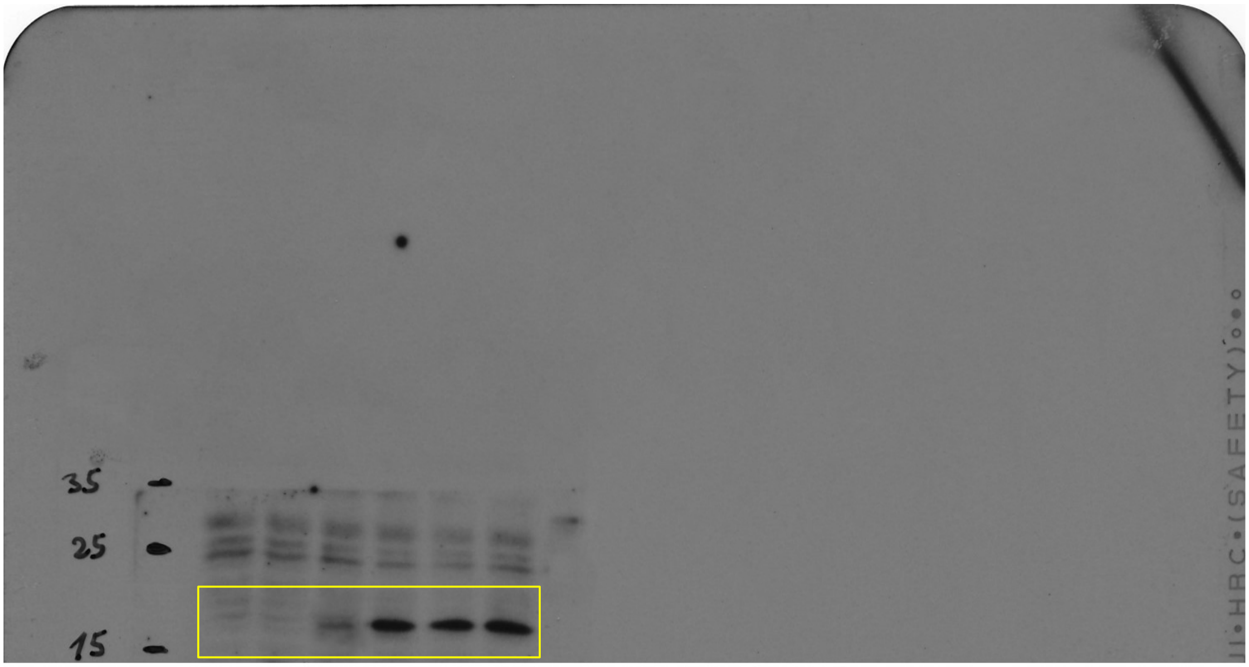

Anti-FTL1

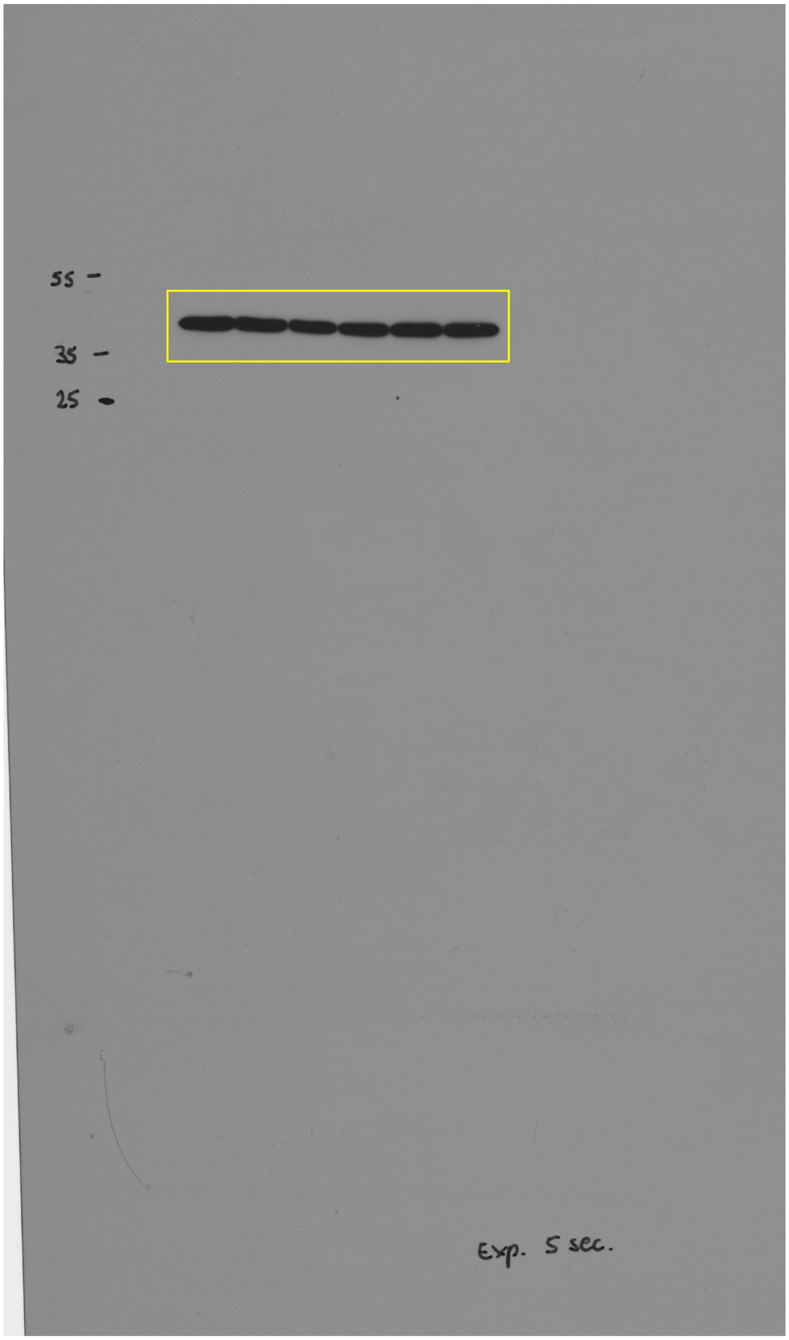

Anti-actin

Full unedited gel for Figure S3C

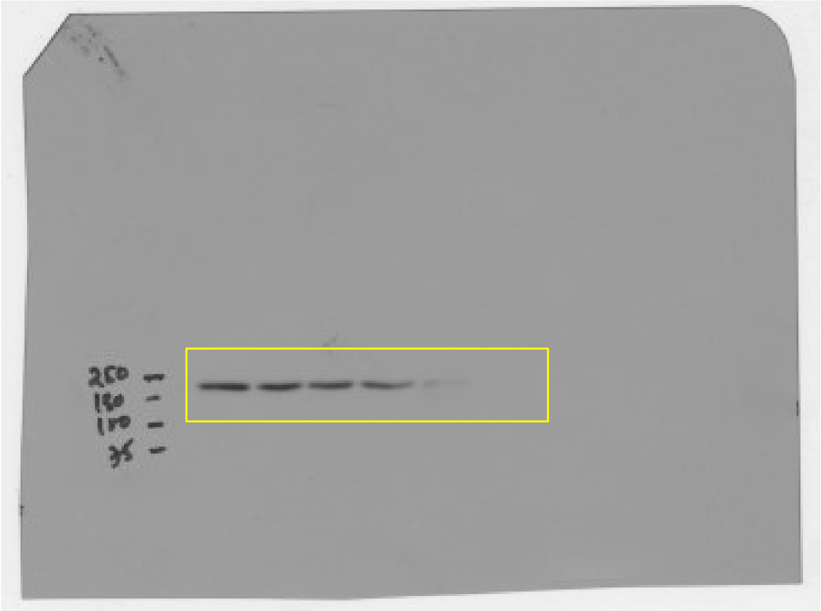

Anti-UBE2O

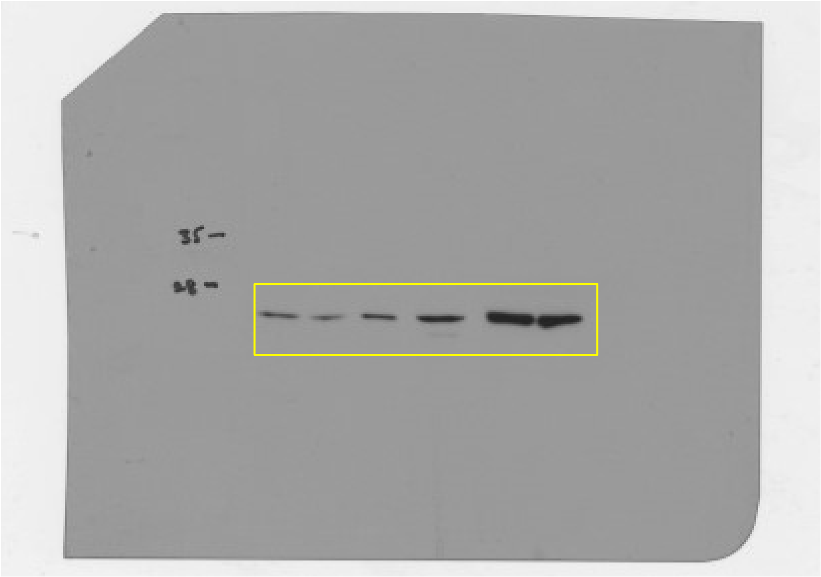

Anti-FTH1

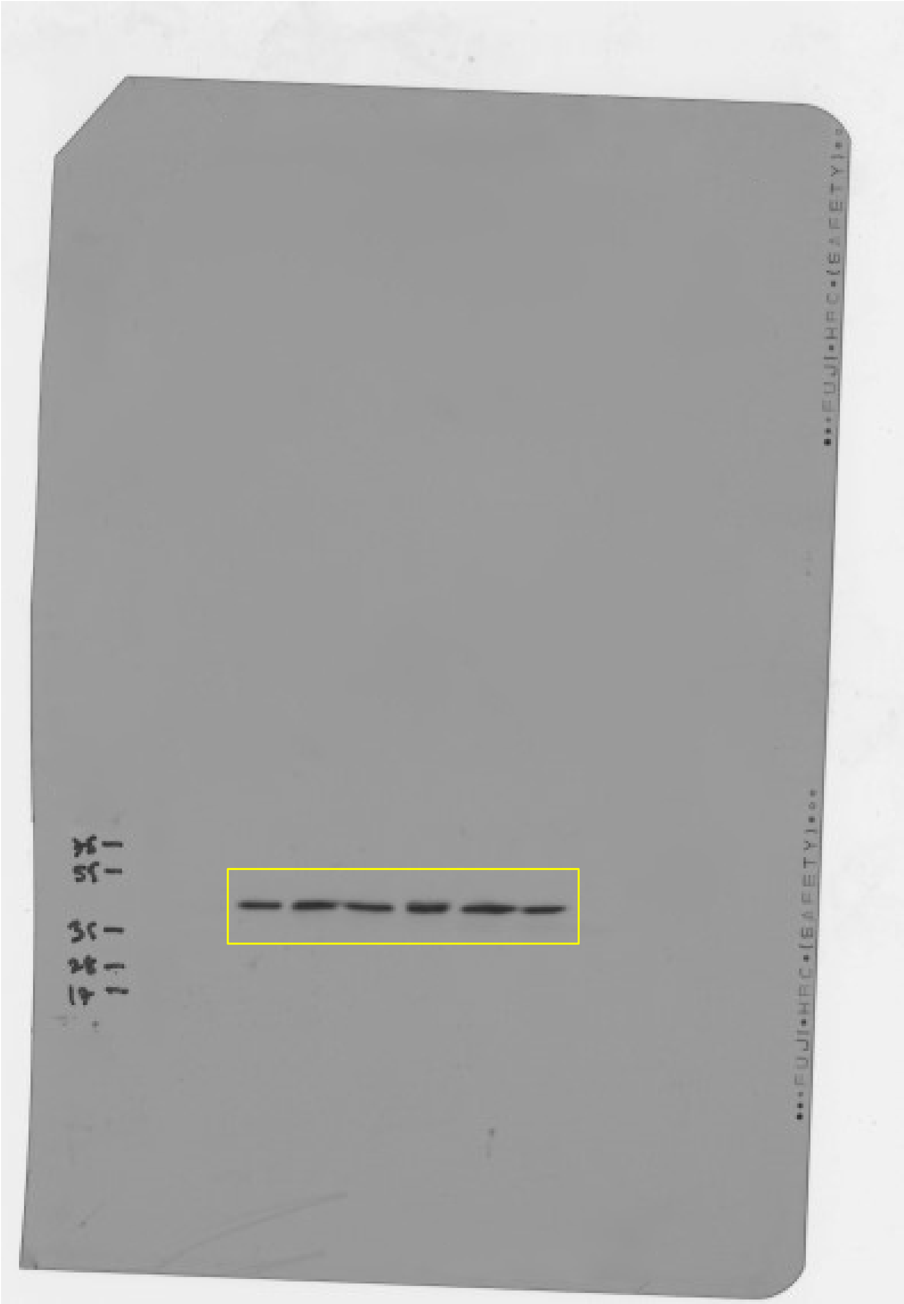

Anti-actin
